# Long-term effects of phosphodiesterase-5 inhibitors on cardiovascular outcomes and death: a systematic review and meta-analysis

**DOI:** 10.1093/ehjcvp/pvae029

**Published:** 2024-07-30

**Authors:** Stergios Soulaidopoulos, Dimitrios Terentes-Printzios, Nikolaos Ioakeimidis, Konstantinos P Tsioufis, Charalambos Vlachopoulos

**Affiliations:** First Cardiology Department, Hippokration Hospital, Athens Medical School, National and Kapodistrian University of Athens, 11527 Athens, Greece; First Cardiology Department, Hippokration Hospital, Athens Medical School, National and Kapodistrian University of Athens, 11527 Athens, Greece; First Cardiology Department, Hippokration Hospital, Athens Medical School, National and Kapodistrian University of Athens, 11527 Athens, Greece; First Cardiology Department, Hippokration Hospital, Athens Medical School, National and Kapodistrian University of Athens, 11527 Athens, Greece; First Cardiology Department, Hippokration Hospital, Athens Medical School, National and Kapodistrian University of Athens, 11527 Athens, Greece

**Keywords:** Erectile dysfunction, Phosphodiesterase 5 inhibitors, Coronary artery disease, Cardiovascular disease, Mortality, Sildenafil

## Abstract

**Aims:**

Phosphodiesterase 5 inhibitors (PDE5i), which are widely used for the treatment of erectile dysfunction (ED), have been found to exhibit systemic vascular benefits by improving endothelial function. In this context, we sought to evaluate the effects of PDE5i on long-term cardiovascular outcomes and mortality.

**Methods and results:**

A comprehensive search of electronic databases was conducted up to 30 May 2023. Cohort studies comparing PDE5i treatment at any dose with other ED treatment, placebo or no treatment and minimum follow-up duration of 6 months were considered eligible. The primary endpoints were: (1) major adverse cardiovascular events (MACE) and (2) all-cause mortality. Pooled risk ratios (RR) with 95% confidence intervals (CI) were calculated. Sixteen studies were included (1 257 759 subjects—10.5% treated with PDE5i). The majority of patients (99.4%) were men [median age 61.5 years (range 30–72.8)]. The median follow-up duration was 4.3 years (range 6 months–7.5 years). PDE5i use was associated with a significant reduction in the composite of MACE (RR 0.78, 95% CI 0.69–0.89). Moreover, the analysis of pooled data from 13 studies, demonstrated that the use of PDE5i was associated with a significantly lower risk of all-cause mortality (RR 0.70, 95% CI 0.56–0.87).

**Conclusion:**

The use of PDE5i primarily in men with or without known coronary artery disease was associated with a lower risk for cardiovascular events and overall mortality. This information underlines that PDE5i could provide clinical benefit beyond ED treatment and could instigate the conduction of further, large-scale randomized clinical trials.

## Graphical Abstract

**Graphical Abstract:**
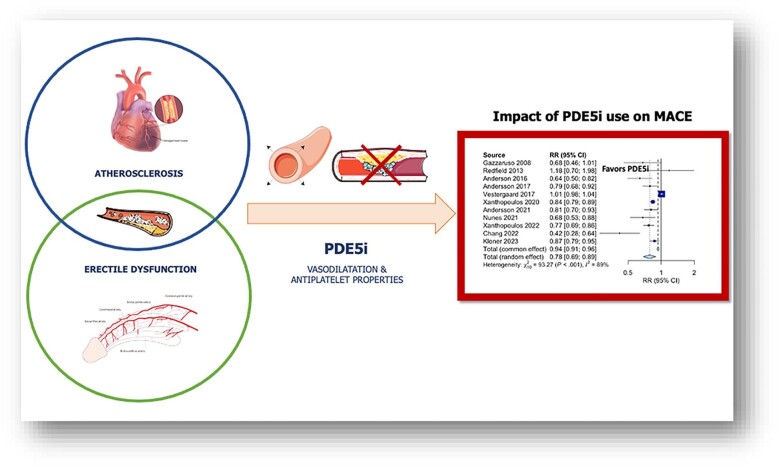
Impact of phosphodiesterase 5 inhibitors on major adverse cardiovascular events. PDE, phosphodisesterase-5 inhibitors; MACE, major adverse cardiovascular events.

## Introduction

Phosphodiesterase 5 inhibitors (PDE5i) constitute a milestone in the treatment of erectile dysfunction (ED). In fact, these drugs were originally developed in the late 1980s for the relief of angina pectoris, with early trials eventually revealing their positive effect on penile erection.^1^ Following the revolution they brought on the field, signalled by the introduction of sildenafil on the market in 1998, the administration of PDE5i was limited for many years to men suffering from ED. Nevertheless, the increasing experience from PDE5i use in ED along with a deeper understanding of cyclic guanosine monophosphate (cGMP)-regulated mechanisms, gradually stimulated the scientific interest for further potential applications of these therapeutic agents. In respect to their mechanism of action, PDE5i work by selectively inhibiting the degradation of cGMP signalling in vascular smooth muscle cells, thereby enhancing nitric-oxide (NO) availability which promotes vascular dilatation.^2^ Apart from their recognized effectiveness in treating ED, PDE5i were found to ameliorate pulmonary vascular resistance and improve several clinical variables in large clinical studies by augmenting NO-mediated vasodilation in the lungs, a finding that led in 2005 to the approval of sildenafil for the treatment of pulmonary arterial hypertension.^3^

Over the last two decades, initial safety concerns have given their place to a growing impression that the use of PDE5i may exhibit several cardiovascular benefits.^4,5^ Starting from animal experimental models, PDE5i were found to attenuate ischaemia—reperfusion myocardial injury and reduce arrhythmia burden, supporting a cardioprotective potential of PDE5i.^6–8^ In human studies, through a combination of direct actions on myocardial tissue and favourable effects on both systemic and pulmonary haemodynamics, PDE5i seem to substantially improve myocardial contractility and clinical variables in patients with systolic heart failure.^9–11^ In the same direction, PDE5i of patients have been shown to reduce pro-inflammatory mediators and improve markers of vascular aging in patients with ED.^12,13^ Recently, though, attention has shifted to the effect of PDE5i on hard cardiovascular endpoints. In particular, accumulating epidemiological data suggest that PDE5i use is probably associated with a lower long-term risk of death and cardiovascular events. These data gain more interest by the fact that they mainly concern patients with ED, which is an established predictor for the development of cardiovascular disease.^14,15^ Nevertheless, not all studies on this field yielded consistent results, while significant diversities in study designs, comparators, drug dosages, and populations exist. Thus, the absolute effect of PDE5i administration on cardiovascular outcomes and death remains still unclear.

Within this framework, the present systematic review and meta-analysis was conducted with the intention to provide an overview of relevant studies and to examine whether and to which extent treatment with PDE5i is associated with a reduction in cardiovascular events and mortality.

## Methods

This systematic review and meta-analysis study was conducted in accordance with the PRISMA (Preferred Reporting Items for Systematic Reviews and Meta-Analyses) guidelines.^16^ The research protocol for this meta-analysis was prospectively registered in the PROSPERO international database (ID: 322288).

### Outcomes

The primary outcomes of interest of this meta-analysis were the following: (i) total number of major adverse cardiovascular events (MACE), including cardiovascular death and nonfatal cardiovascular events (myocardial infarction, ischaemic stroke, revascularization, hospitalization for heart failure, pump thrombosis); and (ii) all-cause mortality. Secondary outcomes were incidence of (i) myocardial infarction and (ii) heart failure.

### Search strategy and selection criteria

A literature search took place in two major databases (PubMed/MEDLINE, Embase) from inception to 30 May 2023, and was restricted to articles published in English. A basic search string using a combination of free text terms and relevant Medical Subject Headings was developed for PubMed and modified accordingly for the other search engines (Supplementary material online). Randomized controlled trials or case-control observational studies evaluating the impact of treatment with PDE5i on hard cardiovascular endpoints and all-cause mortality over a minimum follow-up period of 6 months were considered eligible. Reference lists of the retrieved articles were also screened in order to detect other potentially missed relevant literature. Abstract books of relevant international meetings available online were searched, as well as ClinicalTrials.gov for ongoing relevant studies. We excluded cohort studies not using control groups, studies with a follow-up duration of less than 6 months and those assessing the effect of PDE5i on different outcomes from those defined in our protocol.

### Study selection and data extraction

All retrieved studies were imported into a reference manager software for duplicate removal. Papers were screened by two independent authors (S.S., D.T.P.) for the fulfilment of the inclusion criteria, initially at a title and an abstract level and subsequently by full-text screening of potentially relevant articles. The required data from eligible studies were extracted into a data extraction form, designed according to the Cochrane checklist of items (PICO—Patients, Interventions, Comparisons, Results). All disagreements were resolved by consensus. Numerical data appearing in the selected articles were used. For each of the outcomes of the meta-analysis, adjusted and unadjusted estimates of treatment effects with the 95% confidence intervals (95% CI) as reported in the eligible studies were obtained.

## Study quality assessment

The quality of the included observational studies was evaluated with the Newcastle-Ottawa assessment Scale (NOS) for cohort studies.^17^ The NOS rating system is based on the evaluation of eight quality parameters, which are categorized into three main domains: (1) selection of study groups, (2) comparability of groups, and (3) outcome measurements. The maximum score for each study is 9, with studies scoring less than 5 being considered to exhibit a high risk of bias. The results of the quality assessment of the included studies, except for the RELAX trial which was the only randomized controlled trial, are presented in *Table 2*.

**Table 1.** Main characteristics of the included studies

Study	Study design	Population characteristics	Patients on PDE5i (*N*)	Controls (*n*)	Outcomes	Follow-up (years)
Gazzaruso et al. (2008)	Observational	Men with silent CAD and diabetes	44	74	MACE	47 ± 22 months
Redfield 2013	Randomized controlled study	Patients with preserved heat Failure	113	103	6MWT, HF decompensation, death	24 weeks
Anderson et al. (2016)	Restrospective	Patients with DM type II and elevated CVD risk	1359	4597	Death—myocardial infarction	7.5 years
Andersson et al. (2017)	Restrospective	Patients without prior MI or revascularization	3068	40 077	All cause and CV death—MI-revascularization—prostatectomy—surgery for rectal	3.3 years
Hackett et al. (2017)	Retrospective	Patients with DM type II	175	682	All-cause death	3.8 years
Vestergaard et al. (2017)	Retrospective	Danish men 40–80 years old	71710	992 017	cardiovascular disease, stroke, AMI, ischaemic heart disease, heart failure	3 years
Huang et al. (2020)	Retrospective	Male patients with colorectal cancer	1136	11 329	Death due to CRC, metastasis	4.25 years
Xanthopoulos et al. (2020)	Retrospective	Patients with LVAD	4950	8822	Stroke, LVAD thrombosis, all- cause mortality	48 months
Andersson et al. (2021)	Retrospective	Men with a prior MI or revascularization who received PDE5i or alprostadil (naTve)	16 548	1994	All cause and CV death, HF, MI, PAD, stroke	5.8 years
Nunes et al. (2021)	Retrospective	Patients with erectile dysfunction	3648	3648	Cardiovascular outcomes, death	12 months
Danley 2021	Retrospective	Patients with prostate cancer	1372	1728	All-cause mortality	10 years
Xanthopoulos et al. (2022)	Observational	Patients with LVAD	2173	5056	Stroke, LVAD thrombosis, all- cause mortality	12 ± 8 months
Chang et al. (2022)	Retrospective	Patients with pulmonary hypertension	763	3032	AMI, Ischaemic stroke	7 years
Grandin 2022	Retrospective	Patients with LVAD	1600	1600	Heart failure, all- cause mortality	3 years
Kloner et al. (2023)	Retrospective	Patients with ED without MACE within 1 year	23 816	48 682	All-cause mortality MACE MI heart failure	37 months
Lee et al. (2023)	Retrospective	Patients undergoing Robot-assisted radical prostatectomy	1298	545	All-cause mortality	47 months

CAD, coronary artery disease; CVD, cardiovascular disease; MI, myocardial infarction; PDE5i, phosphodiesterase inhibitors; LVAD, left ventricular assist device; ED, erectile dysfunction; MACE, major adverse cardiovascular events; 6MWT, 6-minute walking test; CV, cardiovascular; AMI, acute myocardial infarction; CRC, colorectal cancer; HF, heart failure; PAD, peripheral arterial disease.

**Table 2:** Newcastle-Ottawa quality assessment scale for observational studies.

	Case-control studies								
	Selection					Exposure			
Studies	Is the case definition adequate	Representa-tivenes of the cases	Selection of controls	Definition of controls	Comparability	Ascertainment of exposure	Same method of ascertainment for cases and controls	Non-response rate	Quality score
Gazzaruso et al. (2008)^23^	*	*	*	*	*	*	*		**7**
**Cohort studies**									
	**Selection**					**Exposure**			
**Studies**	**Representa-tiveness of the exposed cohort**	**Selection of the non-exposed cohort**	**Ascertain-ment of exposure**	**Outcome not present at start of study**	**Comparability based on the design or analysis**	**Assessment of outcome**	**Follow-up long enough**	**Adequacy of follow up of cohorts**	**Quality score**
Anderson et al. (2016)^24^	*	*	*		*	*	*	*	**7**
Andersson et al. (2017)^20^	*	*	*		*	*	*	*	**7**
Hackett et al. (2017)^32^	*	*	*		**	*	*	*	**8**
Vestergaard et al. (2017)^31^	*	*	*		*	*	*	*	**8**
Huang et al. (2020)^33^		*	*		*	*	*		**5**
Xanthopoulos et al. (2020)^35^		*	*		**	*	*	*	**7**
Andersson 2021^21^	*	*	*		*	*	*	*	**7**
Nunes et al. (2021)^34^	*	*	*		**	*	*	*	**8**
Xanthopoulos 2021^36^		*	*		**	*	*	*	7
Danley (2021)^27^	*	*	*		**	*	*	*	8
Grandin (2022)^26^	*	*	*		**	*	*	*	8
Chang et al. (2022)^28^	*	*	*	*	*	*	*	*	8
Kloner et al. (2023)^29^	*	*	*	*	*	*	*	*	8
Lee et al. (2023)^30^	*	*	*		*	*	*	*	7

### Statistical analysis

The summary effects of PDE5i treatment on the endpoints were estimated. The risk ratios (RRs) with 95% CI were initially calculated for individual studies and pooled according to the inverse variance model in order to estimate study weights. A random-effects metanalytic model was selected to obtain pooled estimates of treatment effect with 95% CIs on each of the following outcomes: (1) major cardiovascular events; (2) all-cause mortality; (3) myocardial infarction; (4) heart failure. A separate analysis using the multi-adjusted RRs for each outcome, where applicable, was accordingly performed. Risk estimates reported as hazard ratios, were treated as RRs. A pre-specified subgroup analysis was performed for patients with a history of coronary artery disease (CAD). To quantify heterogeneity across studies, the statistical inconsistency test I^2^ was calculated. The RRs and 95% CIs of individual studies were illustrated with forest plots. The existence of potential publication bias was graphically investigated by funnel plots. A two-tailed *P* value of <0.05 was considered significant. All analyses were performed using the ‘meta’ and ‘metaphor’ packages in the R Project for Statistical Computing (version 3.6.3).

## Results

### Study selection

The combined search of two large databases yielded 1391 unique publications. The preliminary review performed at a title/abstract level identified 36 potentially relevant articles, which were further screened at full-text for eligibility. Of these, 1346 articles were excluded because of absence of a control group (*n* = 1),^18^ duration of follow-up less than 6 months and assessment of endpoints other than cardiovascular events or death, including the effect of PDE5i on surrogate markers of endothelial dysfunction (*n* = 19). Between two publications reporting results for the same cohort at different time points, we selected the one with the longest follow-up period. A recent study by Lagerros et al.,^19^ showing greater overall risk of all-cause mortality (HR: 1.39) as well as higher risk of MACE (HR: 1.70) in patients receiving both nitrate and PDE5i medications, was not included in our analysis since this was published after the end of our literature search, and it would be anyhow rejected since the population used overlapped with two other relevant studies already included in our analysis.^20,21^ Owing to a short follow-up period, a study conducted by Holt et al. concluding that there is neither harm nor benefit from the concomitant use of nitrates and PDE5i, was also deemed ineligible for our analysis.^22^ Sixteen studies evaluating the long-term effects of PDE5i treatment on either cardiovascular outcomes and/or cumulative mortality were ultimately included in the systematic review and in quantitative analysis.^19–34^ Given that only studies with hard cardiovascular endpoints, particularly all-cause mortality, were considered eligible, we did not include pulmonary hypertension trials in this analysis. The PRISMA flow diagram of study selection is depicted in Supplementary material online, *Figure S1* of Supplementary Material 1. The indication for PDE5i use across the studies included in the analysis is demonstrated in Supplementary material online, *Table S1*.

### Characteristics of the included studies

The 16 studies that were selected for the quantitative analysis included a total of 1 257 759 subjects, of whom 132 805 (10.5%) received PDE5i. Regarding their design, 12 of these studies were retrospective studies utilizing health record data to identify patients being prescribed PDE5i,^20–22,24–27,30–34^ while one study retrospectively evaluated follow-up data from 857 diabetic patients that had previously been included in a randomized clinical trial.^32^ Among the eligible studies, there were two prospective observational studies: one aiming to identify predictors of cardiovascular events among 291 diabetic patients with silent CAD^23^ and another examining the association between PDE5i use and outcomes in patients with contemporary centrifugal flow left ventricular assist devices (LVADs).^36^ The RELAX trial, a randomized controlled study evaluating the effect of sildenafil administration for 24 weeks on functional markers of patients with heart failure and preserved ejection fraction, also reported cardiovascular outcomes and was, thus, deemed eligible for our analysis.^25^ The majority (99.4%) of patients included in these studies were men, with a median of age 61.5 years (range 30–72.8), most of them receiving PDE5i for ED. Apart from ED, two studies exclusively included patients with known diabetes, whereas two other included patients with a history of myocardial infarction or coronary revascularization. The presence of both silent CAD and diabetes mellitus was used as a criterion for patient selection in the unique prospective study.^23^ In another study, patients using nitrates with or without PDE5i for ED were selected. Of note, one study investigated the association of PDE5i with the risk of metastasis and all-cause mortality in patients with colorectal cancer^33^ while, in accordance, another one assessed the effect of PDE5i on relapse-free period and overall survival in patients with prostate cancer treated with prostatectomy.^27^ In the same spirit, another study investigated whether the use of PDE5i for ED after robot assisted radical prostatectomy provides a survival benefit in patients with prostate cancer.^30^

Furthermore, three studies reported survival outcomes in patients with LVAD treated with PDE5i.^26,35,36^ Most of the studies were based on prescription drug data to identify patients using PDE5i, but none of the articles provided specific information about the dose and type of the prescribed PDE5i. The follow-up duration ranged between 6 months and 7.5 years across studies. The basic characteristics of the eligible studies that were included in the final analysis are summarized in *Table 1*.

### Major adverse cardiovascular events

Eleven of the included studies evaluated the impact of PDE5i used for the treatment of ED on MACE. The multivariable adjusted RR were pooled for the analysis when provided. Except for the study conducted by Gazzaruso et al.^23^ that dates from 2008, all studies were published between 2013 and 2023 and the mean follow-up ranged from 6 months^34^ to 7.5 years^24^ (median 4.3 years).

**Figure 1.**
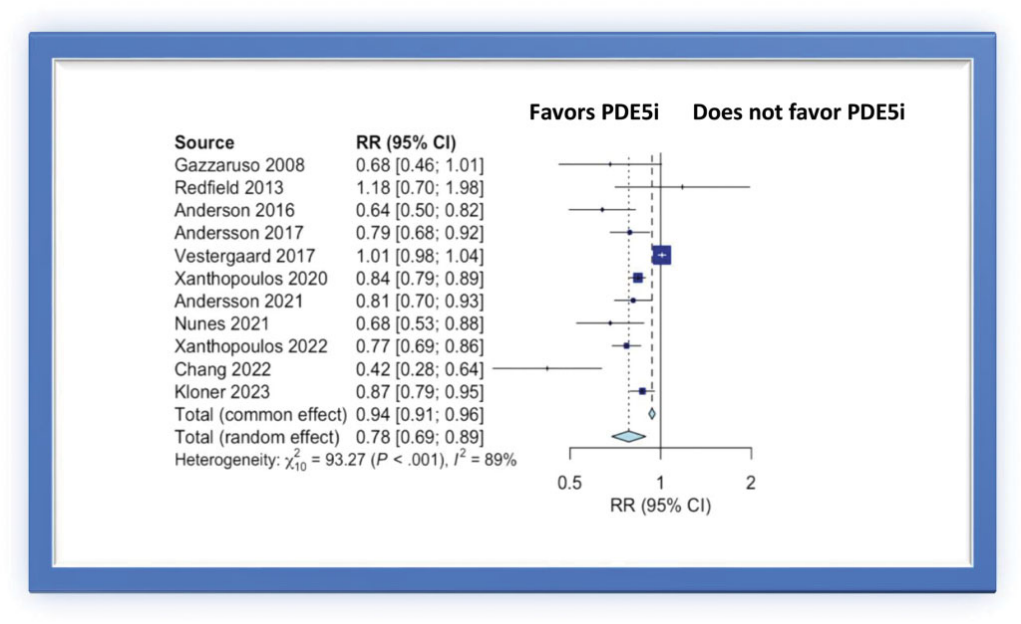
Forest plot of multivariable adjusted RRs of MACE associated with PDE5i use.

The risk of MACE was significantly lower in PDE5i users compared to controls (RR 0.78, 95% CI 0.69–0.89) (*Figure 1*). The *P* value for heterogeneity was <0.001, *I*^2^ = 89%. Even after excluding two studies reporting outcomes of PDE5i administration in patients supported by LVADs,^27,28^ the risk of MACE remained significantly lower in patients that received PDE5i compared to controls (RR 0.77, 95% CI 0.65–0.91, *I*^2^ = 87%–Supplementary material online, *Figure S2*).

Focusing on patients that used PDE5i for ED, including those with a prescription for PDE5i in the large epidemiological registries, the pooled RR for MACE for PDE5i users derived from 7 studies was 0.81 (95% CI 0.72–0.91) compared to controls (Supplementary material online, *Figure S3*).

### All-cause mortality

The outcome of all-cause mortality was evaluated in 12 cohorts. One of these studies evaluated the impact of postoperative administration of PDE5i on cumulative mortality in patients with colorectal cancer and another one in patients with operated prostatic cancer.^27,33^ Three studies included patients on PDE5i for ED,^20,21,24^ while the patients studied by Nunes et al. were taking nitrates with or without PDE5i for ED.^34^ Patients haemodynamically supported by LVADs from three different cohort studies were also included in this analysis.^26,35,36^

**Figure 2.**
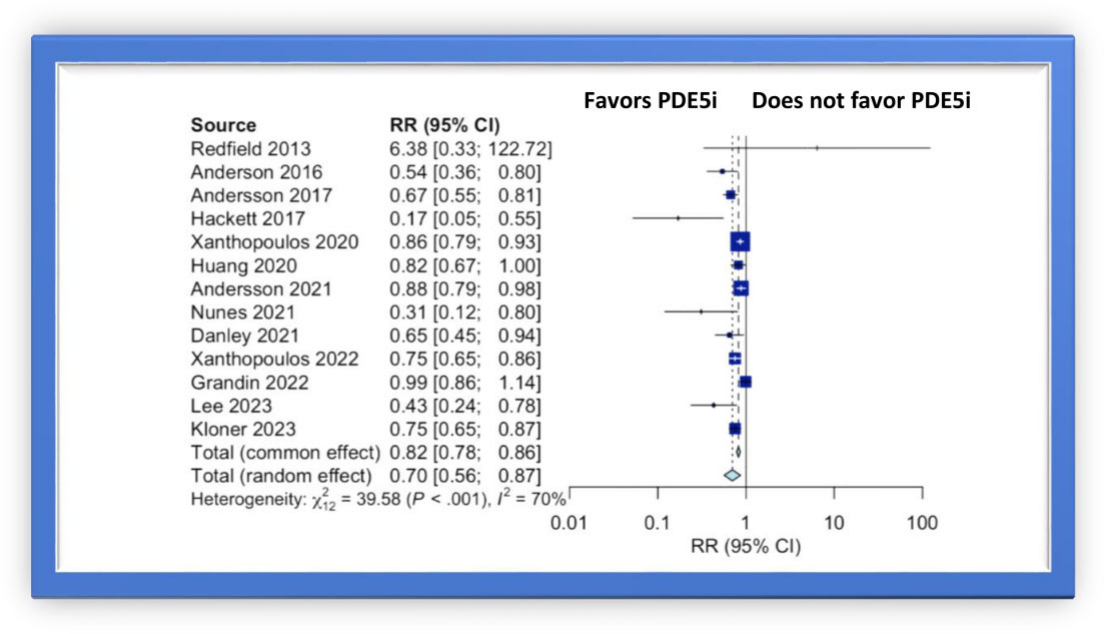
Forest plot of multivariable adjusted RRs of all-cause mortality associated with PDE5i use.

**Figure 3.**
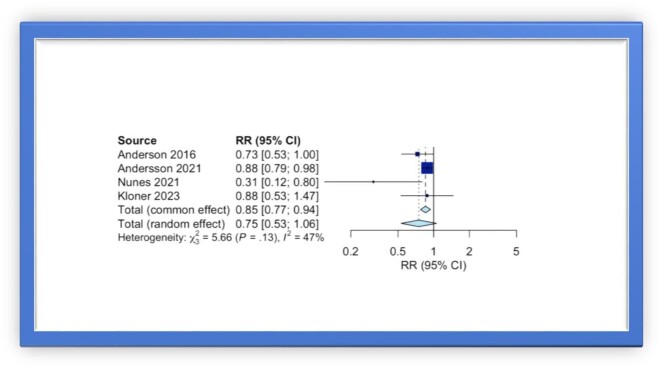
Forest plot of multivariable adjusted RRs of all-cause mortality associated with PDE5i use in patients with a history of coronary artery disease.

In the analysis of pooled data from these 12 studies, the use of PDE5i was associated with a lower risk for all-cause mortality (RR 0.70, 95% CI 0.56–0.87) (*Figure 2*). A sub-analysis focusing on patients with a history of CAD, demonstrated that PDE5i reduce the risk of all-cause mortality by 25% (RR 0.75, 95% CI 0.53–1.06), but this finding was not statistically significant (*Figure 3*).

An additional subgroup analysis based on the indication for PDE5i use was performed. Particularly, studies including patients who received PDE5i for ED were selected. Large epidemiological studies including male patients with a prescription for PDE5i were considered appropriate for this sub-analysis, assuming that the vast majority of these men used PDE5i for ED. The pooled RR for all cause mortality for patients using PDE5i for ED, derived from a total of 9 studies, was 0.63 (95% CI 0.49–0.81) (Supplementary material online, *Figure S4*).

### Myocardial infarction and heart failure

Regarding the secondary outcomes, five studies reported results regarding the effect of PDE5 inhibition on the incidence of myocardial infarction and the analysis of the multivariable adjusted RRs showed a trend towards a statistically significant association between PDE5i treatment and a lower incidence of myocardial infarction (RR 0.78, 95% CI 0.60–1.02) (Supplementary material online, *Figure S5*).^20,21,28,29,31^ Five studies reported results on heart failure and the pooling of results showed that the treatment with PDE5i shows a trend towards reduction of the risk of heart failure (RR 0.84, 95% CI 0.66–1.06) (Supplementary material online, *Figure S6*).^20,21,25,29,31^

## Discussion

This is the first meta-analysis to fully assess the long-term effects of PDE5i on hard cardiovascular endpoints. The major finding of our analysis is that the use of PDE5i is associated with a reduction of 22% in MACE and 30% in all-cause mortality primarily in middle-aged men with a rather elevated baseline risk for cardiovascular events. Interestingly, this benefit seems to be maintained in patients with known, stable CAD.

Indeed, ED represents an independent risk factor for the development of future clinical cardiovascular events.^15^ It is now well established that ED and CAD share mutual vascular risk factors along with common pathogenic features, dominated by vascular endothelial dysfunction.^37,38^ PDE5i restore endothelial function predominantly through the enhancement of nitric oxide viability, inducing the appropriate endothelial-dependent vasorelaxation that is necessary to obtain an erection. Theoretically, owing to their mechanism of action, PDE5i could optimally be suitable for the treatment of cardiovascular disorders. As a matter of fact, prolonged administration of PDE5i in diabetic patients with or without ED has been shown to improve surrogate markers of endothelial dysfunction, such as flow mediated dilatation, and reduce serum indices of vascular inflammation.^12,39,40^ At a clinical level, a generalized improvement of endothelial function promoted by PDE5 inhibition in patients with an elevated cardiovascular risk may be responsible for the substantial decrease in MACE that was observed in our analysis.

Beyond ED, strong evidence demonstrates that PDE5i drastically reduce vascular resistance and improve functional measurements in patients with pulmonary arterial hypertension.^41,42^ Therefore, the treatment of pulmonary arterial hypertension represents for the moment the only established cardiovascular use of PDE5i. In the short term, PDE5i improve survival in patients with pulmonary arterial hypertension, whereas data regarding their impact on cardiovascular events or long-term survival are still lacking.^43^

A number of beneficial effects of PDE5i on heart failure with preserved ejection fraction, have also been reported. These include improvements in pulmonary haemodynamics and left ventricular diastolic function following regression of left ventricular hypertrophy.^44^ In addition, the RELAX trial found that the administration of sildenafil for 6 months in heart failure patients with preserved ejection fraction resulted in a significant improvement in functional capacity and clinical status compared with placebo.^25^

With respect to patients with impaired left ventricular ejection fraction, we identified three studies evaluating the effects of PDE5i administration in patients haemodynamically supported with LVAD. The INTERMACS study recruited more than 7000 patients supported with LVAD, of whom about 2200 received PDE5i after LVAD implantation. PDE5i was associated with lower mortality (adjusted HR: 0.75; 95% CI: 0.65–0.86; *P* < 0.0001) and ischaemic stroke rates (HR: 0.71; 95% CI: 0.56–0.89; *P* < 0.01) at 12 months of follow-up.^36^ In line with these data, the analysis of 13 772 patients with continuous flow LVADs participating in a national registry found a significant association of the postimplant PDE5i administration with a lower risk for thrombotic events (HR, 0.82; 95% CI, 0.74–0.90; *P* < 0.001) and cumulative mortality (HR, 0.86; 95% CI, 0.79–0.93; *P* < 0.001) at 48 months post LVAD implantation.^45^ Although these studies focused on patients with advanced heart failure, who significantly differed from patients with ED that were recruited in most of the eligible cohort studies we decided to include them in our final analysis. These data provide support to the hypothesis that PDE5i may exert antithrombotic effects by potentiating nitric oxide—mediated inhibition of platelet adhesion and aggregation following blockade of cGMP degradation.^46,47^ They also confirm the ability of PDE5i to improve haemodynamics in patients with LVAD, more likely through augmentation of right ventricular function, decrease in pulmonary vascular resistance and improvement of left ventricular filling.^48^

It is noteworthy that the therapeutic potential of PDE5i in colorectal cancer was evaluated in a Swedish cohort included in our analysis.^33^ There is now mounting experimental evidence that inhibition of PDE5 might act against tumour progression and reduce the incidence of metastases among patients with colorectal cancer.^49–51^ While several theories have been proposed, including the induction of apoptosis and effective immune restoration, the potential mechanisms underlying the anti-tumour effect of PDE5i remain largely unclear.^52^ After all, the association of post-diagnostic, post-operative use of PDE5i with a reduced risk of colorectal-specific mortality that was demonstrated in this retrospective study, sets challenging therapeutic targets that worth to be further investigated in randomized clinical trials. This favourable effect of PDE5i on non-cardiovascular mortality could also justify the greater benefit from PDE5 inhibition on all-cause mortality compared to that on MACE that was demonstrated in our analysis.

## Study limitations

We acknowledge that our analysis is not free from certain limitations. First of all, the results of this meta-analysis should be interpreted with caution, taking into consideration that we did not deal with methodological issues of the original studies because they are mainly based on epidemiological data. Differences regarding the primary endpoint, the features of each cohort and the follow-up periods may also account for a certain degree of heterogeneity that was observed across the included studies. Our analysis is further limited by the fact that no dose-response assessment of PDE5i use could be performed, given that such information was not provided or was not available in detail in the eligible publications. Thus, although our analysis indicates a favourable effect of PDE5i on cardiovascular outcomes, the dose of PDE5i that is required to produce a particular effect of interest could not be specified. Another reasonable assumption is that PDE5i use could reflect existing sexual ability, willingness for sexual activity, increased sociability and overall better quality of life, all factors that to some extent may promote increased longevity. Last but not the least, all the eligible cohorts consisted primarily of men, denoting that our results cannot be extrapolated to women.

Eight out of the 10 studies that were included in our analysis recruited patients at high cardiovascular risk or with already known cardiovascular disease. Paradoxically, given that ED was the most common reason for PDE5i use, our analysis demonstrated higher survival rates among patients with ED compared to controls, who were not on PDE5i and thus did not suffer theoretically from ED. The absence of PDE5i use in the control group, on the other hand, does not necessarily exclude the presence of ED, considering that many men with ED do not seek treatment, while none of the included studies evaluated the socioeconomic status, which also seems to be associated with PDE5i use and life expectancy.^53^ It is also possible that PDE5i are protective against cardiovascular disease and, thus, reverse the worse prognosis associated with ED.

## Conclusion

The use of PDE5i for the treatment of ED in male patients at high risk for cardiovascular disease is associated with a substantial reduction in cardiovascular events and rates of overall mortality. The results of this meta-analysis emphatically demonstrate that treatment with PDE5i could provide considerable clinical benefit for specific patient populations beyond the treatment of ED. Whether our findings are potentially applicable to clinical practice is a question that remains to be further elucidated in well-designed, randomized clinical trials.

## Supplementary Material

pvae029_Supplemental_File

## Data Availability

The data underlying this article will be shared on reasonable request to the corresponding author.

## References

[1] Ghofrani HA, Osterloh IH, Grimminger F. Sildenafil: from angina to erectile dysfunction to pulmonary hypertension and beyond. Nat Rev Drug Discov 2006;5:689–702. 16883306 10.1038/nrd2030 PMC7097805

[2] Andersson K-E . PDE5 inhibitors—pharmacology and clinical applications 20 years after sildenafil discovery. Br J Pharmacol 2018;175:2554–2565. 29667180 10.1111/bph.14205 PMC6003652

[3] Galiè N, Ghofrani HA, Torbicki A, Barst RJ, Rubin LJ, Badesch D, Fleming T, Parpia T, Burgess G, Branzi A, Grimminger F, Kurzyna M, Simonneau G. Sildenafil citrate therapy for pulmonary arterial hypertension. N Engl J Med 2005;353:2148–2157. 16291984 10.1056/NEJMoa050010

[4] Kukreja R . Sildenafil and cardioprotection. Curr Pharm Des 2013;19:6842–6847. 23590161 10.2174/138161281939131127110156

[5] Vlachopoulos C, Terentes-Printzios D, Ioakeimidis N, Rokkas K, Stefanadis C. PDE5 Inhibitors in non-urological conditions. Curr Pharm Des 2009;15:3521–3539. 19860698 10.2174/138161209789206980

[6] Maas O, Donat U, Frenzel M, Rütz T, Kroemer HK, Felix SB, Krieg T. Vardenafil protects isolated rat hearts at reperfusion dependent on GC and PKG. Br J Pharmacol 2008;154:25. 18332860 10.1038/bjp.2008.71 PMC2438966

[7] du Toit EF, Rossouw E, Salie R, Opie LH, Lochner A. Effect of Sildenafil on reperfusion function, infarct size, and cyclic nucleotide levels in the isolated rat heart model. Cardiovasc Drugs Ther 2005 191 2005;19:23–31. 15883753 10.1007/s10557-005-6894-2

[8] Salloum FN, Abbate A, Das A, Houser J-E, Mudrick CA, Qureshi IZ, Hoke NN, Roy SK, Brown WR, Prabhakar S, Kukreja RC. Sildenafil (Viagra) attenuates ischemic cardiomyopathy and improves left ventricular function in mice. Am J Physiol—Hear Circ Physiol 2008;294. H1398–H1406. 10.1152/ajpheart.91438.2007 18223185

[9] Hutchings DC, Anderson SG, Caldwell JL, Trafford AW. Phosphodiesterase-5 inhibitors and the heart: compound cardioprotection? Heart 2018;104:1244–1250. 29519873 10.1136/heartjnl-2017-312865 PMC6204975

[10] Lewis GD, Shah R, Shahzad K, Camuso JM, Pappagianopoulos PP, Hung J, Tawakol A, Gerszten RE, Systrom DM, Bloch KD, Semigran MJ. Sildenafil improves exercise capacity and quality of life in patients with systolic heart failure and secondary pulmonary hypertension. Circulation 2007;116:1555–1562. 17785618 10.1161/CIRCULATIONAHA.107.716373

[11] Hirata K, Adji A, Vlachopoulos C, O'rourke MF. Effect of sildenafil on cardiac performance in patients with heart failure. Am J Cardiol 2005;96:1436–1440. 16275194 10.1016/j.amjcard.2005.06.091

[12] Vlachopoulos C, Ioakeimidis N, Rokkas K, Angelis A, Terentes-Printzios D, Stefanadis C, Tousoulis D. Acute effect of sildenafil on inflammatory markers/mediators in patients with vasculogenic erectile dysfunction. Int J Cardiol 2015;182:98–101. 25577741 10.1016/j.ijcard.2014.12.072

[13] Vlachopoulos C, Terentes‐Printzios D, Ioakeimidis N, Rokkas K, Samentzas A, Aggelis A, Kardara D, Stefanadis C. Beneficial effect of vardenafil on aortic stiffness and wave reflections. J Clin Pharmacol 2012;52:1215–1221. 21953573 10.1177/0091270011413586

[14] Terentes-Printzios D, Ioakeimidis N, Rokkas K, Vlachopoulos C. Interactions between erectile dysfunction, cardiovascular disease and cardiovascular drugs. Nat Rev Cardiol 2022;19:59–74. 34331033 10.1038/s41569-021-00593-6

[15] Vlachopoulos C, Jackson G, Stefanadis C, Montorsi P. Erectile dysfunction in the cardiovascular patient. Eur Heart J 2013;34:2034–2046. 23616415 10.1093/eurheartj/eht112

[16] Hutton B, Salanti G, Caldwell DM, Chaimani A, Schmid CH, Cameron C, Ioannidis JP, Straus S, Thorlund K, Jansen JP, Mulrow C, Catalá-López F, Gøtzsche PC, Dickersin K, Boutron I, Altman DG, Moher D. The PRISMA extension statement for reporting of systematic reviews incorporating network meta-analyses of health care interventions: checklist and explanations. Ann Intern Med 2015;162:777–784. https://doi.org/107326/M14-2385 26030634 10.7326/M14-2385

[17] Wells G, Shea B, O'Connell D, Peterson J, Welch V, Losos M, Tugwell P, Wells Ga S, Zello G, Petersen J. The Newcastle-Ottawa Scale (NOS) for assessing the quality of nonrandomised studies in meta-analyses. Available from: http://www.ohri.ca/programs/clinical_epidemiology/oxford.htm (last accessed 5 May 2023)

[18] Mittleman MA, Maclure M, Lewis MA, Hall GC, Moore N, Giuliano F, Porst H, Hedelin H, Martin-Morales A, Sobel RE, Reynolds R, Glasser DB. Cardiovascular outcomes among sildenafil users: results of the International Men's Health Study. Int J Clin Pract 2008;62:367–373. 18261073 10.1111/j.1742-1241.2007.01679.x

[19] Trolle Lagerros Y, Grotta A, Freyland S, Grannas D, Andersson DP. Risk of death in patients with coronary artery disease taking nitrates and phosphodiesterase-5 inhibitors. J Am Coll Cardiol 2024;83:417–426. 38233015 10.1016/j.jacc.2023.10.041

[20] Andersson DP, Trolle Lagerros Y, Grotta A, Bellocco R, Lehtihet M, Holzmann MJ. Association between treatment for erectile dysfunction and death or cardiovascular outcomes after myocardial infarction. Heart 2017;103:1264–1270. 28280146 10.1136/heartjnl-2016-310746 PMC5537549

[21] Andersson DP, Landucci L, Lagerros YT, Grotta A, Bellocco R, Lehtihet M, Holzmann MJ. Association of phosphodiesterase-5 inhibitors versus Alprostadil with survival in men with coronary artery disease. J Am Coll Cardiol 2021;77:1535–1550. 33766260 10.1016/j.jacc.2021.01.045

[22] Holt A, Blanche P, Jensen AKG, Nouhravesh N, Rajan D, Jensen MH, El-Sheikh M, Schjerning A-M, Schou M, Gislason G, Torp-Pedersen C, Mcgettigan P, Lamberts M. Adverse events associated with coprescription of phosphodiesterase type 5 inhibitors and oral organic nitrates in male patients with ischemic heart disease A case-crossover study. Ann Intern Med 2022;175:774–782. 35436155 10.7326/M21-3445

[23] Gazzaruso C, Solerte SB, Pujia A, Coppola A, Vezzoli M, Salvucci F, Valenti C, Giustina A, Garzaniti A. Erectile dysfunction as a predictor of cardiovascular events and death in diabetic patients with angiographically proven asymptomatic coronary artery disease. A potential protective role for statins and 5-phosphodiesterase inhibitors. J Am Coll Cardiol 2008;51:2040–2044. 18498958 10.1016/j.jacc.2007.10.069

[24] Anderson SG, Hutchings DC, Woodward M, Rahimi K, Rutter MK, Kirby M, Hackett G, Trafford AW, Heald AH. Phosphodiesterase type-5 inhibitor use in type 2 diabetes is associated with a reduction in all-cause mortality. Heart 2016;102:1750–1756. 27465053 10.1136/heartjnl-2015-309223 PMC5099221

[25] Redfield MM, Chen HH, Borlaug BA, Semigran MJ, Lee KL, Lewis G, Lewinter MM, Rouleau JL, Bull DA, Mann DL, Deswal A, Stevenson LW, Givertz MM, Ofili EO, O'connor CM, Felker GM, Goldsmith SR, Bart BA, Mcnulty SE, Ibarra JC, Lin G, Oh JK, Patel MR, Kim RJ, Tracy RP, Velazquez EJ, Anstrom KJ, Hernandez AF, Mascette AM, Braunwald E, RELAX Trial. Effect of phosphodiesterase-5 inhibition on exercise capacity and clinical status in heart failure with preserved ejection fraction: a randomized clinical trial. JAMA 2013;309:1268–1277. 23478662 10.1001/jama.2013.2024 PMC3835156

[26] Grandin EW, Gulati G, Nunez JI, Kennedy K, Rame JE, Atluri P, Pagani FD, Kirklin JK, Kormos RL, Teuteberg J, Kiernan MS. Outcomes with phosphodiesterase-5 inhibitor use after left ventricular assist device: an STS-INTERMACS analysis. Circ Hear Fail 2022;15:E008613. 10.1161/CIRCHEARTFAILURE.121.008613 PMC9205418 35332780

[27] Danley KT, Tan A, Catalona WJ, Leikin R, Helenowski I, Jovanovic B, Gurley M, Kuzel TM. The association of phosphodiesterase-5 inhibitors with the biochemical recurrence-free and overall survival of patients with prostate cancer following radical prostatectomy. Urol Oncol Semin Orig Investig 2022;40:57.e1–57.e7. 10.1016/j.urolonc.2021.05.031 34284930

[28] Chang W-T, Su C-C, Chang Y-C, Cheng C-L, Hsu C-H. The impact of Sildenafil on ischemic outcomes in patients with pulmonary hypertension—A nationwide cohort study. Acta Cardiol Sin 2022;38:623–630. 36176365 10.6515/ACS.202209_38(5).20220401A PMC9479051

[29] Kloner RA, Stanek E, Crowe CL, Singhal M, Pepe RS, Bradsher J, Rosen RC. Effect of phosphodiesterase type 5 inhibitors on major adverse cardiovascular events and overall mortality in a large nationwide cohort of men with erectile dysfunction and cardiovascular risk factors: a retrospective, observational study based on healthc. J Sex Med 2023;20:38–48. 36897243 10.1093/jsxmed/qdac005

[30] Lee J, Kim HR, Heo JE, Jang WS, Lee KS, Kang SK, Han H, Choi YD. Phosphodiesterase-5 inhibitor use in robot assisted radical prostatectomy patients is associated with reduced risk of death: a propensity score matched analysis of 1,058 patients. World J Mens Health 2023;41:1–8. 36649919 10.5534/wjmh.220063 PMC10523119

[31] Vestergaard N, Søgaard P, Torp-Pedersen C, Aasbjerg K. Relationship between treatment of erectile dysfunction and future risk of cardiovascular disease: a nationwide cohort study. Eur J Prev Cardiol 2017;24:1498–1505. 28656785 10.1177/2047487317718082

[32] Hackett G, Jones PW, Strange RC, Ramachandran S. testosterone and phosphodiesterase 5-inhibitor treatments and age related mortality in diabetes. World J Diabetes 2017;8:104. 28344753 10.4239/wjd.v8.i3.104 PMC5348622

[33] Huang W, Sundquist J, Sundquist K, Ji J. Phosphodiesterase-5 inhibitors use and risk for mortality and metastases among male patients with colorectal cancer. Nat Commun 2020;11: 3191. 32581298 10.1038/s41467-020-17028-4 PMC7314744

[34] Nunes AP, Seeger JD, Stewart A, Gupta A, Mcgraw T. Cardiovascular outcome risks in patients with erectile dysfunction Co-prescribed a phosphodiesterase type 5 inhibitor (PDE5i) and a nitrate: a retrospective observational study using electronic health record data in the United States. J Sex Med 2021;18:1511–1523. 34389264 10.1016/j.jsxm.2021.06.010

[35] Xanthopoulos A, Tryposkiadis K, Triposkiadis F, Fukamachi K, Soltesz EG, Young JB, Wolski K, Blackstone EH, Starling RC. Postimplant phosphodiesterase type 5 inhibitors use is associated with lower rates of thrombotic events after left ventricular assist device implantation. J Am Heart Assoc 2020;9: e015897 32648508 10.1161/JAHA.119.015897 PMC7660717

[36] Xanthopoulos A, Wolski K, Wang Q, Blackstone EH, Randhawa VK, Soltesz EG, Young JB, Nissen SE, Estep JD, Triposkiadis F, Starling RC. Postimplant phosphodiesterase-5 inhibitor use in centrifugal flow left ventricular assist devices. JACC Hear Fail 2022;10:89–100. 10.1016/j.jchf.2021.09.008 35115092

[37] Vlachopoulos C, Aznaouridis K, Ioakeimidis N, Rokkas K, Vasiliadou C, Alexopoulos N, Stefanadi E, Askitis A, Stefanadis C. Unfavourable endothelial and inflammatory state in erectile dysfunction patients with or without coronary artery disease. Eur Heart J 2006;27:2640–2648. 17056702 10.1093/eurheartj/ehl341

[38] Viigimaa M, Vlachopoulos C, Doumas M, Wolf J, Imprialos K, Terentes-Printzios D, Ioakeimidis N, Kotsar A, Kiitam U, Stavropoulos K, Narkiewicz K, Manolis A, Jelakovic B, Lovic D, Kreutz R, Tsioufis K, Mancia G. Update of the position paper on arterial hypertension and erectile dysfunction. J Hypertens 2020;38:1220–1234. 32073535 10.1097/HJH.0000000000002382

[39] Aversa A, Vitale C, Volterrani M, Fabbri A, Spera G, Fini M, Rosano GMC. Chronic administration of Sildenafil improves markers of endothelial function in men with type 2 diabetes. Diabet Med 2008;25:37–44. 18199130 10.1111/j.1464-5491.2007.02298.x

[40] Santi D, Granata ARM, Guidi A, Pignatti E, Trenti T, Roli L, Bozic R, Zaza S, Pacchioni C, Romano S, Nofer JR, Rochira V, Carani C, Simoni M. Six months of daily treatment with vardenafil improves parameters of endothelial inflammation and of hypogonadism in male patients with type 2 diabetes and erectile dysfunction: a randomized, double-blind, prospective trial. Eur J Endocrinol 2016;174:513–522. 26792933 10.1530/EJE-15-1100

[41] Wilkins MR, Wharton J, Grimminger F, Ghofrani HA. Phosphodiesterase inhibitors for the treatment of pulmonary hypertension. Eur Respir J 2008;32:198–209. 18591337 10.1183/09031936.00124007

[42] Barnes H, Brown Z, Burns A, Williams T. Phosphodiesterase 5 inhibitors for pulmonary hypertension. Cochrane Database Syst Rev 2019;1: CD012621. 30701543 10.1002/14651858.CD012621.pub2 PMC6354064

[43] Zeng W‐J, Sun Y‐J, Gu Q, Xiong C‐M, Li J‐J, He J‐G. Impact of Sildenafil on survival of patients with idiopathic pulmonary arterial hypertension. J Clin Pharmacol 2012;52:1357–1364. 21956607 10.1177/0091270011418656

[44] Guazzi M, Vicenzi M, Arena R, Guazzi MD. PDE5 inhibition with sildenafil improves left ventricular diastolic function, cardiac geometry, and clinical status in patients with stable systolic heart failure: result of a 1-year, prospective, randomized, placebo-controlled study. Circ Hear Fail 2011;4:8–17. 10.1161/CIRCHEARTFAILURE.110.944694 21036891

[45] Xanthopoulos A, Tryposkiadis K, Triposkiadis F, Fukamachi K, Soltesz EG, Young JB, Wolski K, Blackstone EH, Starling RC. Postimplant phosphodiesterase type 5 inhibitors use is associated with lower rates of thrombotic events after left ventricular assist device implantation. J Am Heart Assoc 2020;9:e015897. 32648508 10.1161/JAHA.119.015897 PMC7660717

[46] Andersson K‐E . PDE5 inhibitors—pharmacology and clinical applications 20 years after sildenafil discovery. Br J Pharmacol 2018;175:2554. 29667180 10.1111/bph.14205 PMC6003652

[47] Zayat R, Ahmad U, Stoppe C, Khattab MA, Arab F, Moza A, Tewarie L, Goetzenich A, Autschbach R, Schnoering H. Sildenafil reduces the risk of thromboembolic events in HeartMate II patients with low-level hemolysis and significantly improves the pulmonary circulation. Int Heart J 2018;59:1227–1236. 30305587 10.1536/ihj.18-001

[48] Nagendran J, Archer SL, Soliman D, Gurtu V, Moudgil R, Haromy A, St. Aubin C, Webster L, Rebeyka IM, Ross DB, Light PE, Dyck JRB, Michelakis ED. Phosphodiesterase type 5 is highly expressed in the hypertrophied human right ventricle, and acute inhibition of phosphodiesterase type 5 improves contractility. Circulation 2007;116:238–248. 17606845 10.1161/CIRCULATIONAHA.106.655266

[49] Peak TC, Richman A, Gur S, Yafi FA, Hellstrom WJG. The role of PDE5 inhibitors and the NO/cGMP pathway in cancer. Sex Med Rev 2016;4:74–84. 27872007 10.1016/j.sxmr.2015.10.004

[50] Islam BN, Sharman SK, Hou Y, Bridges AE, Singh N, Kim S, Kolhe R, Trillo-Tinoco J, Rodriguez PC, Berger FG, Sridhar S, Browning DD. Sildenafil suppresses inflammation-driven colorectal cancer in mice. Cancer Prev Res 2017;10:377–388. 10.1158/1940-6207.CAPR-17-0015 PMC5530733 28468928

[51] Mei XL, Yang Y, Zhang YJ, Li Y, Zhao JM, Qiu JG, Zhang WJ, Jiang QW, Xue YQ, Zheng DW, Chen Y, Qin WM, Wei MN, Shi Z. Sildenafil inhibits the growth of human colorectal cancer in vitro and in vivo. Am J Cancer Res 2015;5:3311–3324. 26807313 PMC4697679

[52] Pantziarka P, Sukhatme V, Crispino S, Bouche G, Meheus L, Sukhatme VP. Repurposing drugs in oncology (ReDO)—selective PDE5 inhibitors as anti-cancer agents. Ecancermedicalscience 2018;12:824 29743944 10.3332/ecancer.2018.824 PMC5931815

[53] Travison TG, Hall SA, Fisher WA, Araujo AB, Rosen RC, Mckinlay JB, Sand MS. Correlates of PDE5i use among subjects with erectile dysfunction in two population-based surveys. J Sex Med 2011;8:3051–3057. 21834873 10.1111/j.1743-6109.2011.02423.x PMC3548233

