# Supplementary material for: Long-term effects of phosphodiesterase-5 inhibitors on cardiovascular outcomes and death: a systematic review and meta-analysis
Source: Eur Heart J Cardiovasc Pharmacother. 2024 Jul 30;10(5):403–12. doi: 10.1093/ehjcvp/pvae029 (PMC11323371; doi:10.1093/ehjcvp/pvae029)

**Search terms**

30/05/2023, 21:00

**MEDLINE search strategy via pubmed**

1. phosphodiesterase type 5 inhibitor (9513)

2. sildenafil (8154)

3. Viagra (8334)

4. Revatio (8156)

5. tadalafil (2438)

6. Cialis (2473)

7. Adcirca (2439)

8. vardenafil (1232)

9. Levitra (1243)

10. Staxyn (1233)

11. erectile dysfunction (27,021)

12. 1 OR 2 OR 3 OR 4 OR 5 OR 6 OR 7 OR 8 OR 9 OR 10 OR 11 (33,412)

13. myocardial infarction (287,846)

14. death (1,077,350)

15. cardiovascular death (151,013)

16. heart failure (292,927)

17. all-cause mortality (51,780)

18. 12 OR 13 OR 14 OR 15 OR 16 OR 17 (1,519,964)

19. 12 AND 18 (1,660)

20. animal (7,202,883)

21. 19 NOT 20 (1,389)

((((((((((((((sildenafil) OR (sildenafil)) OR (Viagra)) OR (Revatio)) OR (tadalafil)) OR (cialis)) OR (vardenafil)) OR (Adcirca)) OR (levitra)) OR (Staxyn))) OR (erectile dysfunction))) AND (((((myocardial infarction) OR (death)) OR (cardiovascular death)) OR (heart failure)) OR (all-cause mortality))) NOT (animal)

**Supplemental Figure 1.** Summary of evidence search and selection


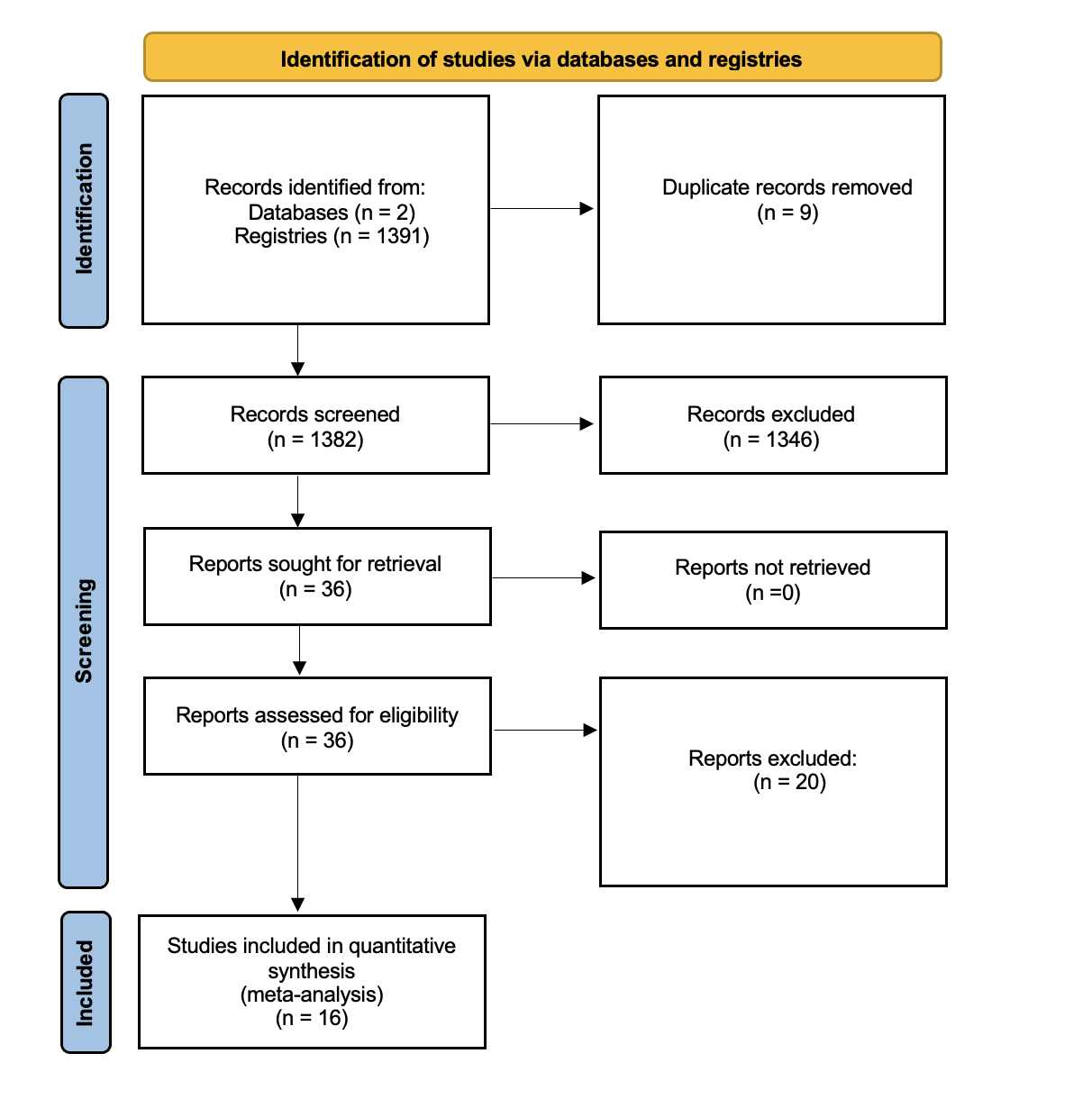


**Supplemental Table 1.** Indications for PDE5i among the studies included in the analysis.

*
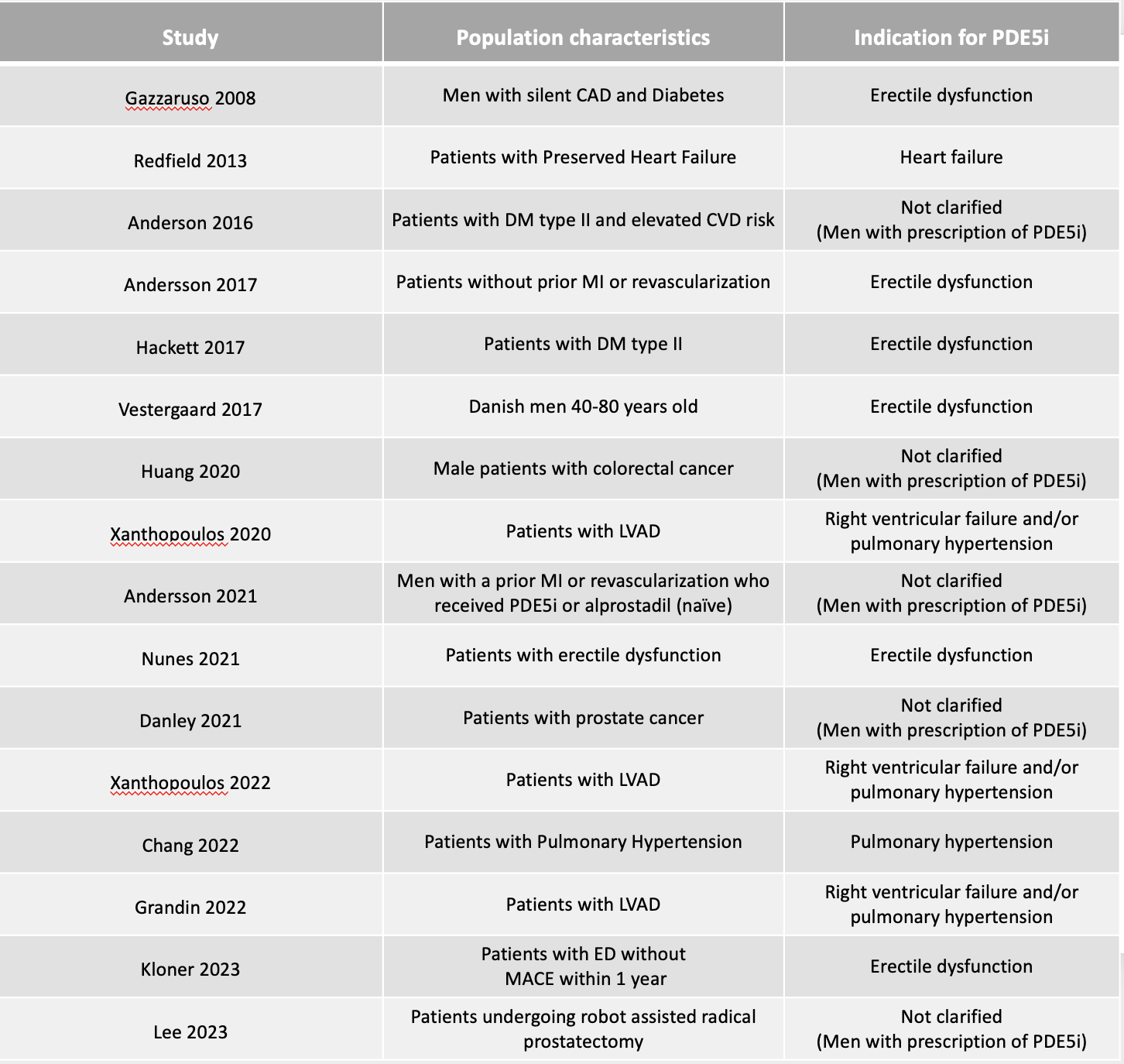
*

CAD, coronary artery disease; CVD, cardiovascular disease; MI, myocardial infarction; PDE5i, phosphodiesterase inhibitors; LVAD, left ventricular assist device; ED, erectile dysfunction; MACE, major adverse cardiovascular events.

**Supplemental Figure 2.** Forest plot of multivariable adjusted RRs for major adverse cardiovascular events associated with PDE5i use after exclusion of LVAD studies

**
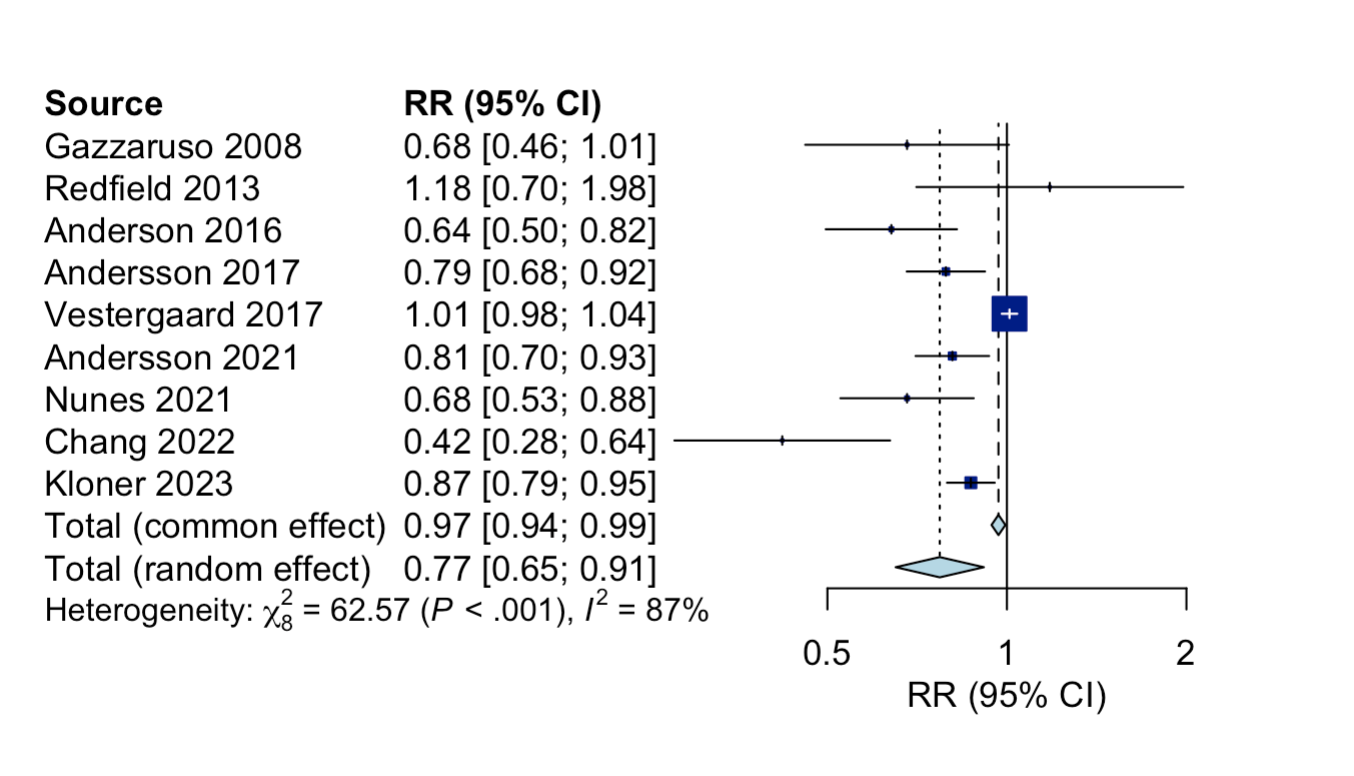
**

**Supplemental Figure 3.** Forest plot of multivariable adjusted RRs for major adverse cardiovascular events associated with PDE5i use with an indication of erectile dysfunction

**
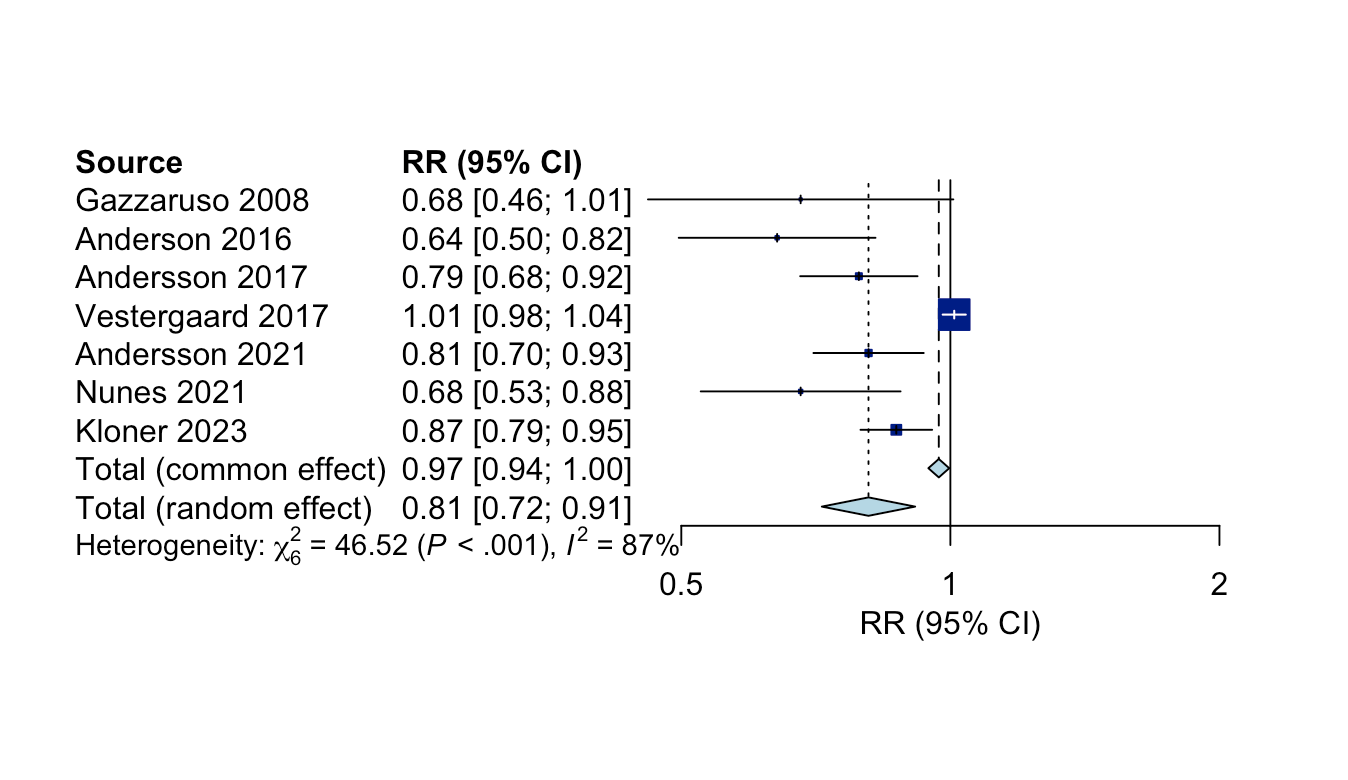
**

**Supplemental Figure 4.** Forest plot of multivariable adjusted RRs of all-cause mortality associated with PDE5i use for erectile dysfunction


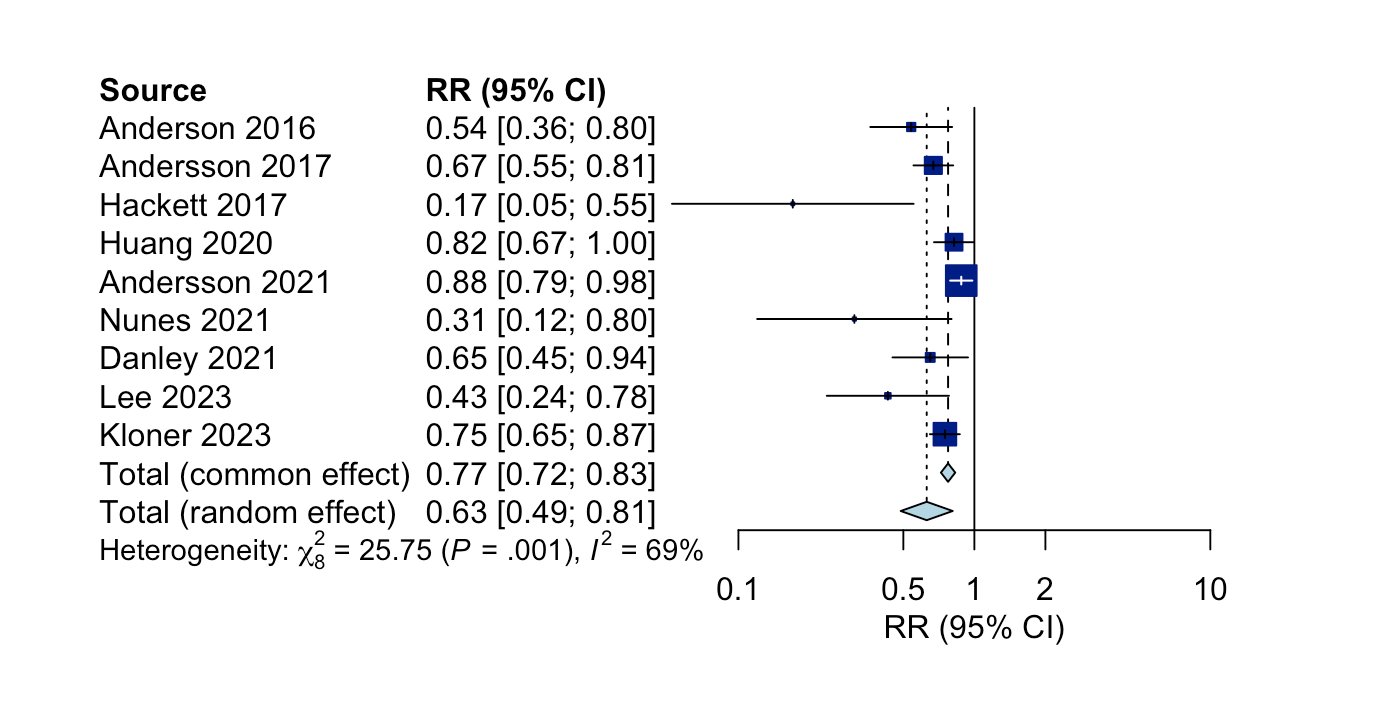


**Supplemental Figure 5.** Forest plot of multivariable adjusted RRs for myocardial infarction associated with PDE5i use


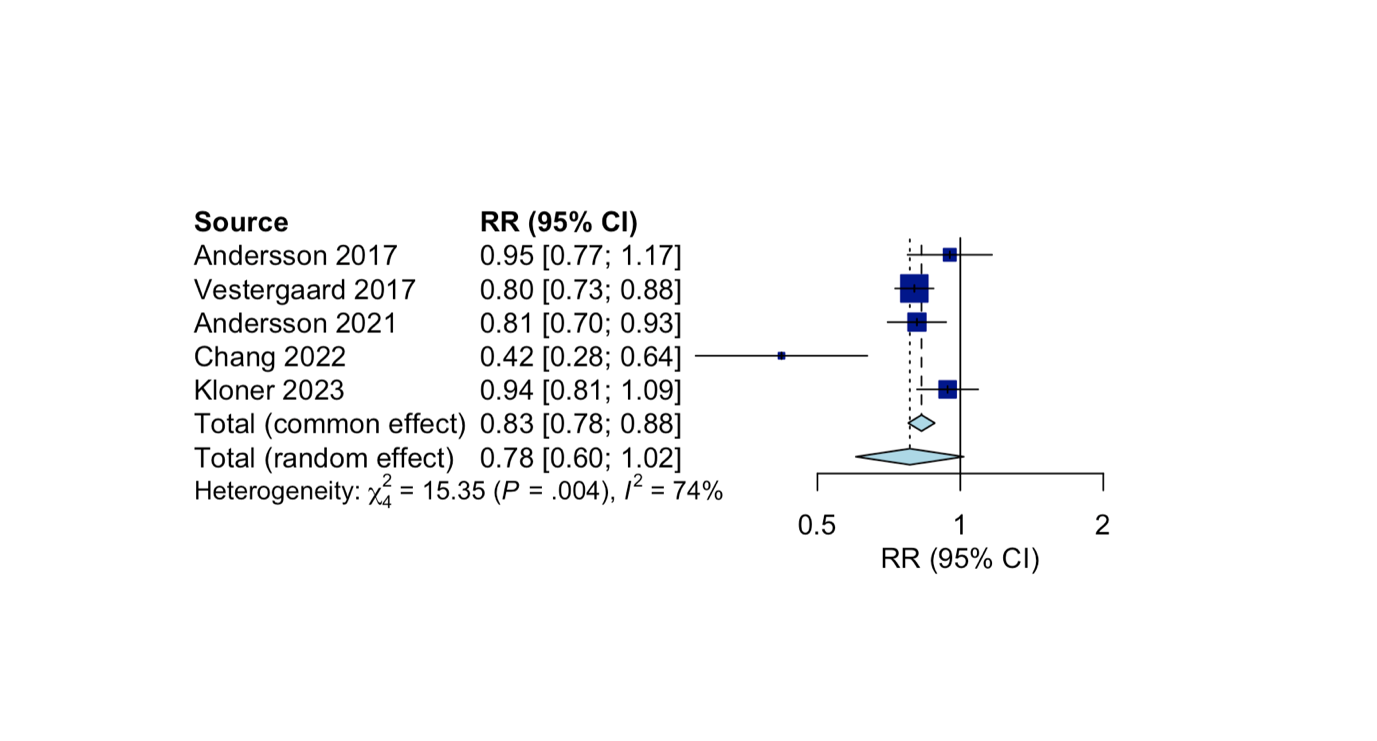


**Favors PDE5i Does not favor PDE5i**

**Supplemental Figure 6.** Forest plot of multivariable adjusted RRs for heart failure associated with PDE5i use


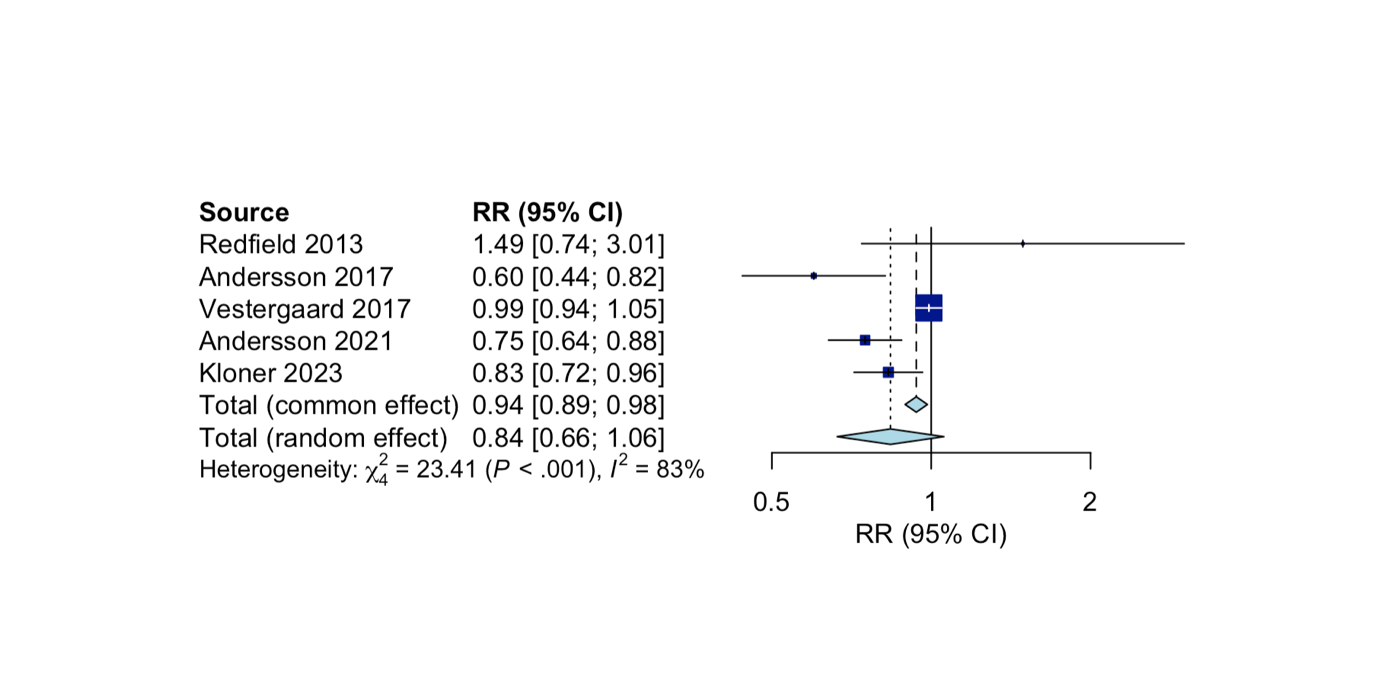


**Favors PDE5i Does not favor PDE5i**

**Supplemental figure 5.** Funnel plot for studies included in the analysis for major adverse cardiovascular events


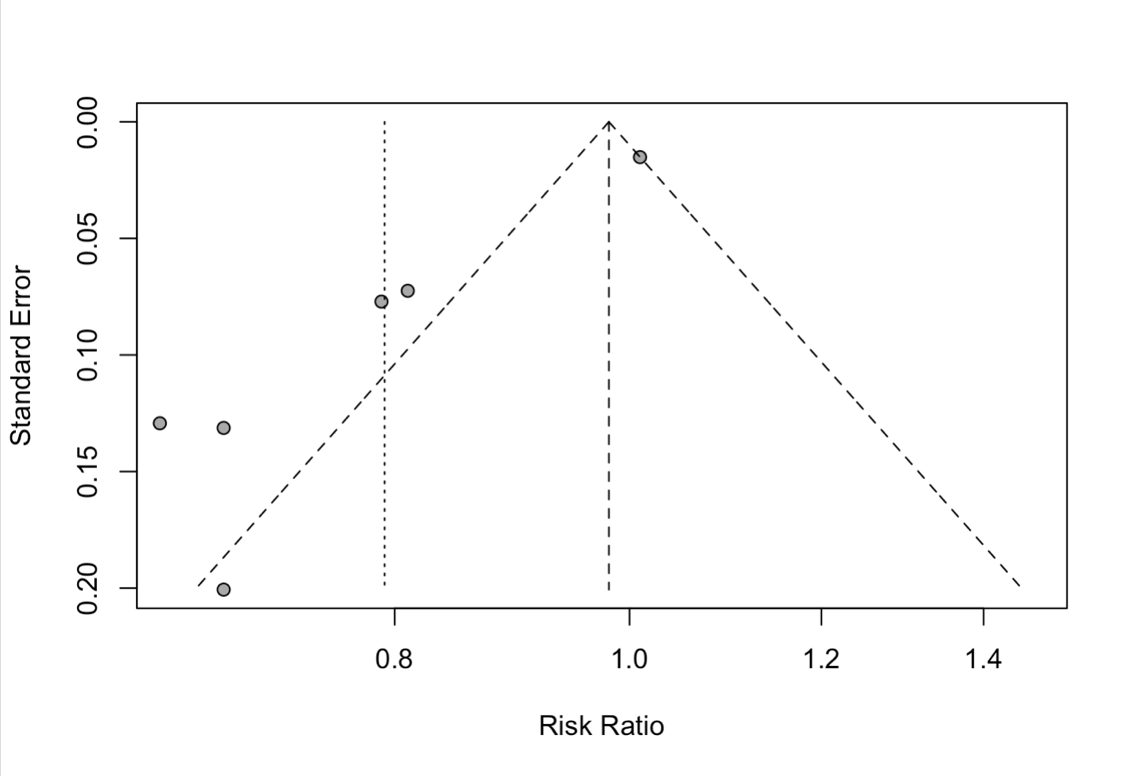


**Supplemental figure 6.** Funnel plot for studies included in the analysis for all-cause mortality.


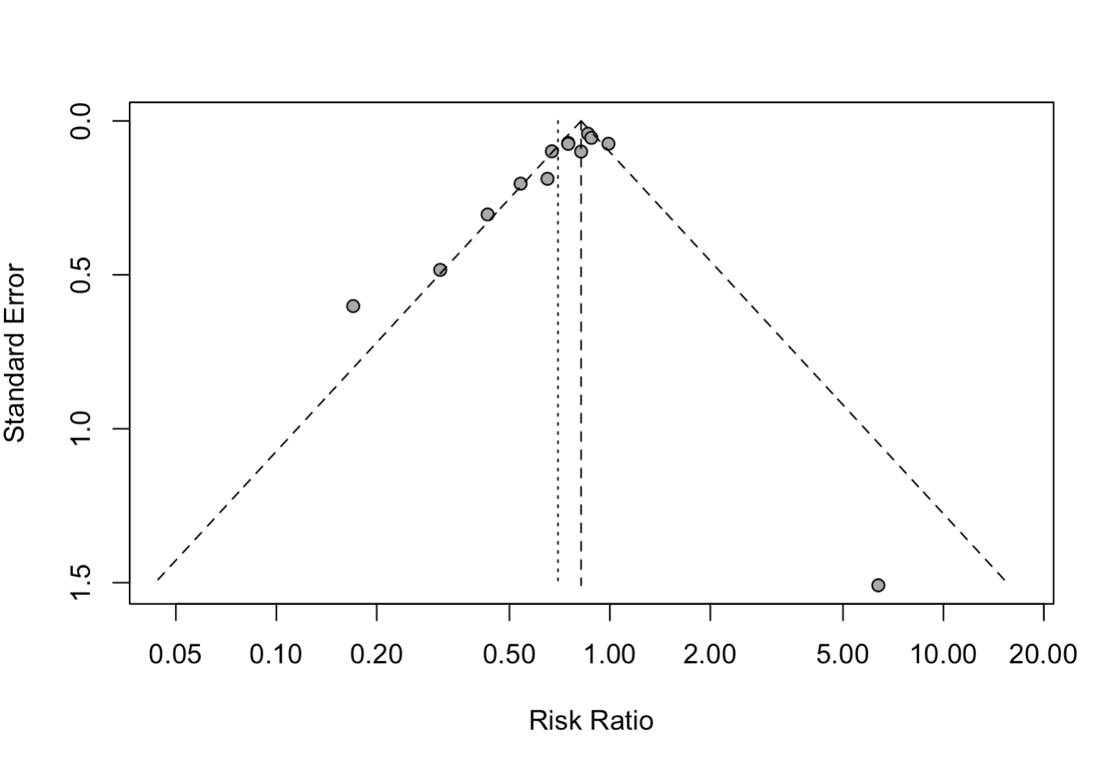

Supplement: pvae029_Supplemental_File [file pvae029_supplemental_file.docx]
